# Assessing the Status Quo of EHR Accessibility, Usability, and Knowledge Dissemination

**DOI:** 10.5334/egems.228

**Published:** 2018-05-25

**Authors:** Saif Khairat, George Cameron Coleman, Samantha Russomagno, David Gotz

**Affiliations:** 1 University of North Carolina, US

**Keywords:** EHR, Accessibility, Usability, Knowledge, Dissemination

## Abstract

**Aim:**

This study was performed to better characterize accessibility to electronic health records (EHRs) among informatics professionals in various roles, settings, and organizations across the United States and internationally.

**Background:**

The EHR landscape has evolved significantly in recent years, though challenges remain in key areas such as usability. While patient access to electronic health information has gained more attention, levels of access among informatics professionals, including those conducting usability research, have not been well described in the literature. Ironically, many informatics professionals whose aim is to improve EHR design have restrictions on EHR access or publication, which interfere with broad dissemination of findings in areas of usability research.

**Methods:**

To quantify the limitations on EHR access and publication rights, we conducted a survey of informatics professionals from a broad spectrum of roles including practicing clinicians, researchers, administrators, and members of industry. Results were analyzed and levels of EHR access were stratified by role, organizational affiliation, geographic region, EHR type, and restrictions with regard to publishing results of usability testing, including screenshots.

**Results:**

126 respondents completed the survey, representing all major geographic regions in the United States. 71.5 percent of participants reported some level of EHR access, while 13 percent reported no access whatsoever. Rates of no-access were higher among faculty members and researchers (19 percent). Among faculty members and researchers, 72 percent could access the EHR for usability and/or research purposes, but, of those, fewer than 1 in 3 could freely publish screenshots with results of usability testing and half could not publish such data at all. Across users from all roles, only 21 percent reported the ability to publish screenshots freely without restrictions.

**Conclusions:**

This study offers insight into current patterns of EHR accessibility among informatics professionals, highlighting restrictions that limit dissemination of usability research and testing. Further conversations and shared responsibility among the various stakeholders in industry, government, health care organizations, and informatics professionals are vital to continued EHR optimization.

## Introduction

By now, more than 94 percent of U.S. hospitals have adopted a certified electronic health record (EHR) system [1]. While digitization was touted as a way to improve health care quality and access and reduce cost, the net effects have been mixed [2]. The ultimate dream of intuitive EHR systems that empower end-users and improve patient safety remains unmet. Usability is a critical element of this vision that has not advanced at a sufficient pace.

Medical errors and adverse events related to EHR usability and/or design have been identified for years [2]. In addition, the misuse of electronic health records was recently identified as the top threat to patient safety for health care organizations [3]. Although the Office of the National Coordinator for Health Information Technology (ONC) describes EHR usability as a priority [4], policy-based roadblocks and contractual restrictions enforced by major vendors currently limit the degree to which usability research can be freely disseminated. Furthermore, while other industries such as aviation, transportation, and engineering have incorporated vigorous usability testing as a means to improve safety, studies have shown limited use of user-centered design principles from EHR vendors [5]. In addition to these barriers, many usability researchers and scientists often have limited EHR access to facilitate usability testing. While EHR access status has been described among medical student [6] and remote clinician populations [7], to our knowledge there have been no prior reports of EHR access status among the broader population of informatics professionals, which importantly includes a subset of usability researchers.

To better understand the relationship between EHR usability and accessibility, we conducted a survey of members of the American Medical Informatics Association community, including clinicians, researchers and scientists, and administrative personnel. The purpose of this paper is to quantify the limitations on EHR access status and publication rights among informatics professionals. We present the current state of EHR accessibility among clinical and non-clinical users, which to the best of our knowledge has not been previously examined in the literature. We also provide recommendations for changes in culture and policy as they relate to an overarching goal of improved health information technology (health IT) and patient safety.

### The Risk to Patient Safety

Even before the digitization of our health care system, the impact of medical errors on patient safety was well-described [8]. In general, health IT has been recognized for its direct link to patient safety [9] and poor usability or design of health IT has been identified as an important culprit in medical errors [10], including examples related to medication bar coding [11], physician order entry [12], and other safety domains [13]. More recently, safety concerns have been linked to EHRs in particular, specifically as they relate to workflow and data display [14].

EHRs are a major source of data for clinicians and researchers. Massive volumes of clinical data—structured and unstructured data, from an increasing number of sources—are readily available, but it is the synthesis of these electronic data that is key to effective care delivery. Unfortunately, the electronic deluge can impair a clinician’s cognitive capacity through information overload and alert fatigue [15,16].

In an environment of continued errors and adverse events stemming from EHR systems that are either misused or poorly designed, usability research has emerged as a key bridge to a safer future. However, advances in the field of usability research have not seen significant industry penetration.

### Policy Roadblocks

The ONC’s 2014 Standards and Certification Criteria included safety-enhanced design certification requirements. Under these criteria, vendors were expected to implement a user-centered design as well as conduct and report the results of usability testing on eight EHR functions (e.g., e-prescribing, clinical decision support, medication and allergy lists, etc.) [17]. Nonetheless, Ratwani et al. found that despite these requirements, significant variability remains with regard to vendor design and incorporation of user-centered design principles [18].

Interestingly, the policy landscape has been in flux recently, with new changes to vendor certification requirements purportedly to reduce burdens on development and improve interoperability [19]. Despite these changes, major barriers to EHR optimization remain. Among these, many leading EHR vendors impose restrictions on the publication of screenshots and other material essential to communicating the results of usability research. Vendors argue that screenshots represent their company’s intellectual property. Ironically, even the vendors’ restrictive publication policies themselves are sometimes considered confidential. As one Chief Medical Informatics Officer at a major academic medical center in the Midwest reported: “[Our vendor] has a document that describes the limitations [on publishing screenshots]. They ask to approve all screenshots prior to publication.” However, “I’m not allowed to share the document describing the limitations.” These restrictions are legally enforced, as restrictions are often embedded within formal contracts and terms of use. One investigation of 11 contracts between EHR vendors and major health systems in New York, California, and Florida revealed that 10 of the 11 contained a clause protecting large disclosures of information from public exposure [20]. Thus, the current climate reflects a formalized system of censorship, which significantly hinders usability testing.

Despite growing emphasis on patient engagement and efforts to increase patient access to the EHR [21], less attention has been paid to increasing EHR access among non-clinical members of the informatics community. While patient confidentiality concerns justify restricted EHR access to a certain extent, there is still a subset of faculty members and researchers interested in usability who cannot access the EHR to conduct research, even in a training environment without confidential patient information. The patient safety implications of EHR publication censorship and restricted EHR access are multiple. First, limiting institutions from sharing usability research findings can prevent the correction of known problems. Second, without public dissemination, poor design practices will propagate to future iterations of existing vendor systems. Finally, research efforts are directed away from real-world usability problems at a time when EHR systems have become widely deployed and when an urgency exists to accelerate usability testing. Indeed, a 2011 Institute of Medicine report identified contractual restrictions as a barrier to knowledge regarding patient safety risks related to health IT [9,20].

## Methods

### Sample

We surveyed approximately 3000 members of the American Medical Informatics Association (AMIA) regarding their access to EHR systems. The survey was fielded over a 4-month span wherein a questionnaire was disseminated via the discussion forums of selected AMIA Working Groups (WG). We targeted those WG’s associated with keywords as below, demonstrating robust conversation among members about EHR challenges and barriers: “Implementation,” “Clinical Research Information Systems,” “Clinical Information Systems,” “Nursing Informatics,” “Intensive Care Informatics,” “Visual Analytics,” and “Global Health Informatics.” The survey questionnaire was designed, administered, and collected through the Google Forms tool.

Inclusion criteria were defined as practicing clinicians, researchers, administrative leaders (e.g., Chief Medical Information Officers), and full-time professionals with an informatics role. Student entries were excluded. A total of 132 responses were collected, of which 126 were used in the final analysis after the aforementioned exclusion criteria were applied. Survey results were automatically transformed into a database and exported into a CSV file for further statistical analysis.

### Survey Tool

**Figure 1:**
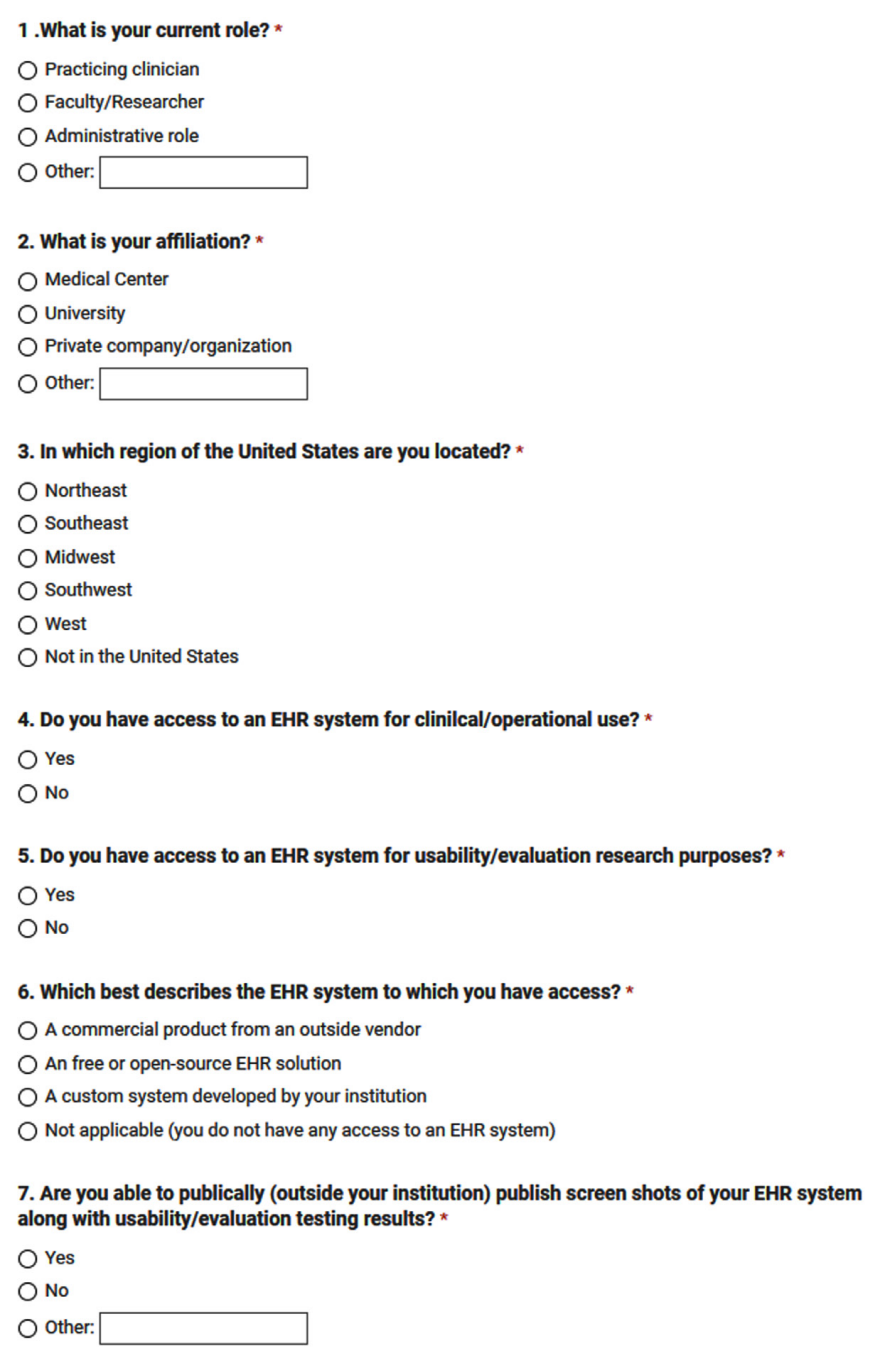
Usability Survey.

The EHR Usability survey questions were informed by literature (Figure 1) [5]. The survey did not collect personal information and did not allow for unique identification of participants. The survey was comprised of seven items designed to characterize key information among respondents: current role (e.g., clinician, researcher, administration, other), affiliation (e.g., medical center, university, private organization, other), geographic region (Northeast, Southeast, Midwest, Southwest, West, outside the United States), access to EHR for clinical and/or operational use, access to EHR for research and/or usability purposes, type of EHR (commercial, open-source, self-developed), and the ability to externally publish screenshots of the EHR along with results of usability evaluation and testing. Multiple-choice items were utilized to reduce data discrepancy or inaccuracy. Some questions did include a response for “Other” which allowed for free-response input. As shown in Figure 1, all survey responses were discreet data since questions were either categorical (1–4) or binary (0–1). Survey data was imported, organized, and coded in Microsoft Excel and then exported to SPSS statistical software and Tableau, a data visualization software, for further analysis.

## Results

**Figure 2:**
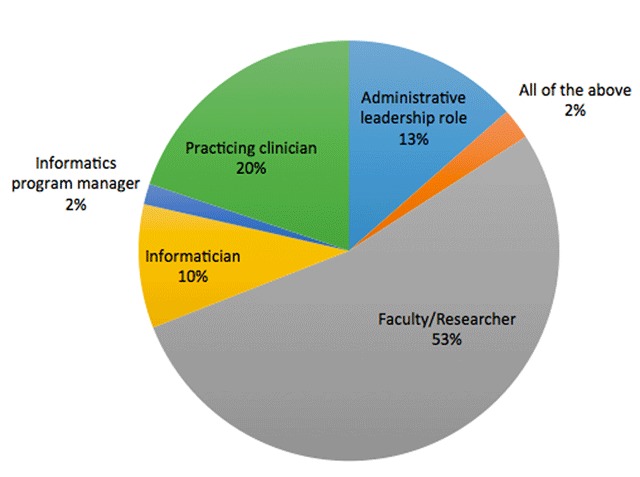
Characterization of Participants by Professional Role.

The sample (n = 126) consisted of individuals in the informatics community with a range of professional roles (Table 1). Approximately half of respondents (53 percent) were faculty members or researchers involved with informatics research, followed by practicing clinicians with informatics roles (20 percent) (Figure 2). Approximately 15 percent of participants served in administrative leadership positions with roles such as Chief Medical Information Officers (CMIO), and approximately 10 percent of participants were from industry with roles such as consultants or informatics specialists. Three participants reported more than one role.

**Table 1:** EHR Access and Publication Status Stratified by Professional Role.

		Clinicians		Faculty/Research		Admin/Leadership		Industry		Project Manager		All the Above		TOTAL	

		n = 25 20%		n = 67 53%		n = 17 13%		n = 12 12%		n = 2 2%		n = 3 2%		n = 126	

**Access Status**	**Clinical/Operational**	25	100%	36	54%	16	94%	8	67%	2	100%	3	100%	90	71%
	**Usability/Research**	11	44%	48	72%	13	76%	7	58%	2	100%	3	100%	84	67%
	**No access**	0	0%	13	19%	1	6%	3	25%	0	0%	0	0%	17	13%

**EHR Status**	**Vendor**	23	92%	44	66%	13	76%	8	67%	1	50%	3	100%	92	73%
	**Open source**	1	4%	5	7%	1	6%	0	0%	0	0%	0	0%	7	6%
	**In-house**	1	4%	9	13%	2	12%	1	8%	1	50%	0	0%	14	11%
	**None**	0	0%	9	13%	1	6%	3	25%	0	0%	0	0%	13	10%

**Publication Status**	**Yes**	6	24%	15	22%	4	24%	0	0%	1	50%	1	33%	27	21%
	**Yes, with permission**	0	0%	6	9%	0	0%	1	8%	1	50%	1	33%	9	7%
	**Unsure**	5	20%	3	4%	1	6%	2	17%	0	0%	0	0%	11	9%
	**No**	14	56%	43	64%	12	71%	9	75%	0	0%	1	33%	79	63%

**Figure 3:**
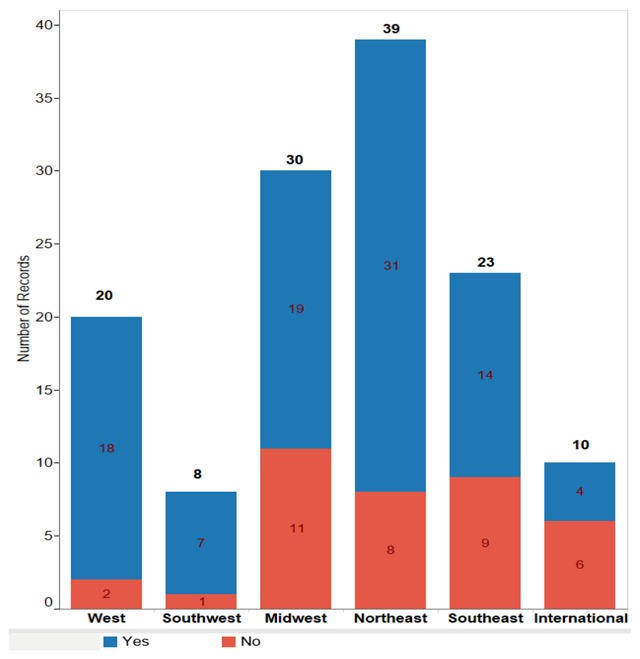
Breakdown of Participants With and Without Access by Geographic Region.

Geographically, most participants (92 percent) were from the U.S., with the remainder working internationally. All U.S. regions were represented, with the highest distribution of U.S. respondents from the Northeast region (approximately one-third) and the smallest distribution from the Southwest region (6 percent) (Figure 3).

### Access Status

**Figure 4:**
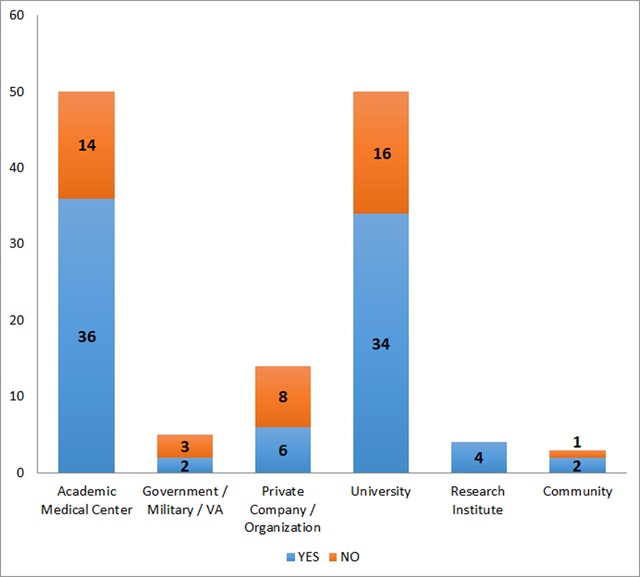
Characterization of EHR Access by Professional Association.

EHR accessibility among respondents was assessed. Levels of access were determined separately for clinical and/or operational use and for usability and/or evaluation purposes. Overall, the majority of respondents (71 percent) reported having access to the main EHR at their institution for clinical and/or operational use, including 100 percent of practicing clinicians with EHR access. Nearly three-fourths of all respondents (73 percent) reported EHR access for usability and/or evaluation purposes, including 76 percent access among researchers and faculty members. However, across users from all roles, there was a 13 percent rate of no-access to the EHR (i.e. no access for clinical/operational use or usability/evaluation purposes). Among faculty members/researchers, rates of no-access were higher (19 percent). Our data also revealed variation in rates of EHR access across affiliations, with higher rates at private research institutes and among participants with dual appointments but lower rates among industry, medical centers, and government employees (Figure 4).

### EHR Status

**Figure 5:**
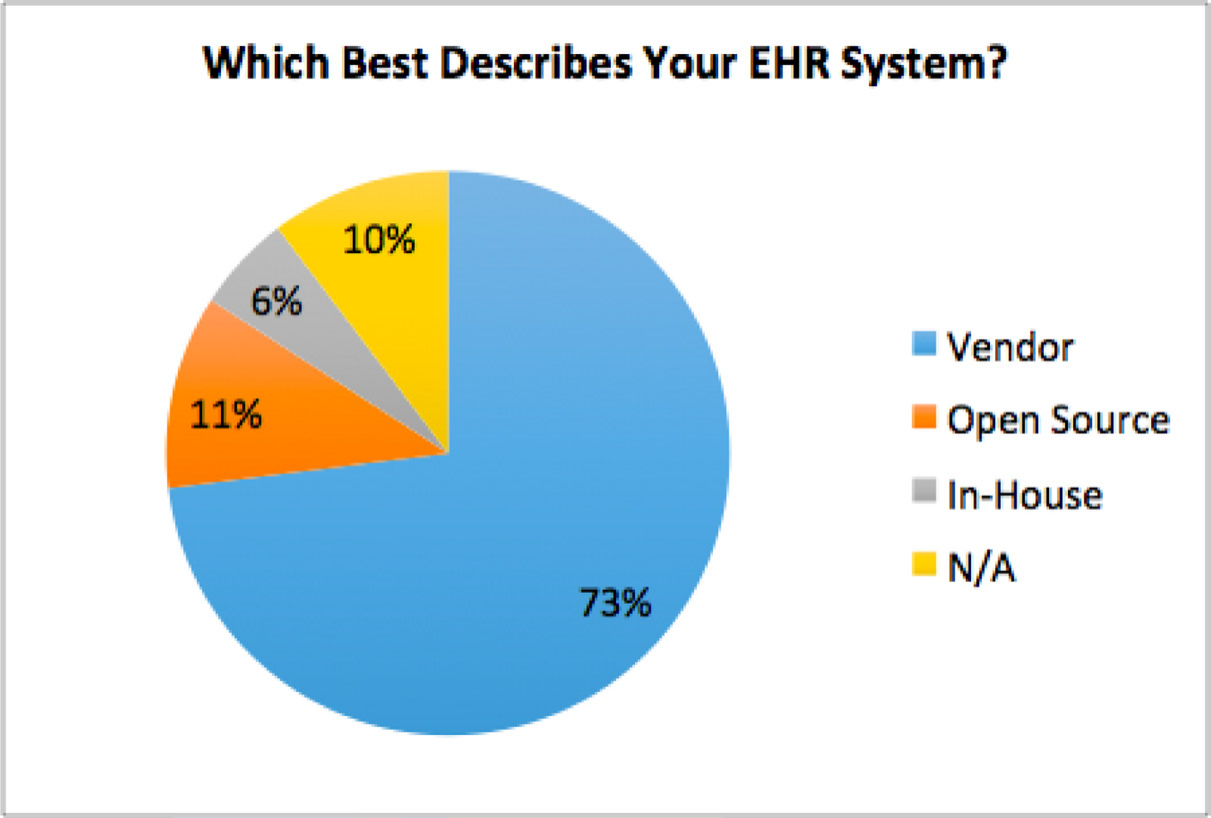
Trends in EHR Types Reported.

Nearly three out of four participants (73 percent) reported use of a commercial EHR developed by an external vendor, 10 percent reported an in-house EHR that was developed by their institution, and only 6 percent reported a free, open-source EHR (Figure 5).

### Dissemination Status

Dissemination status was assessed by determining whether participants were able to publish public screenshots of their EHR (i.e. outside their institution) along with usability and/or evaluation testing results. In total, 21 percent of respondents reported the ability to disseminate screenshots publicly without special permission, while an additional 7 percent reported the ability to disseminate with special permission and 9 percent of participants were unsure of any restrictions on publication of screenshots. However, the majority of participants (63 percent) reported that they did not have rights to publish screenshots of the EHR for research purposes.

**Figure 6:**
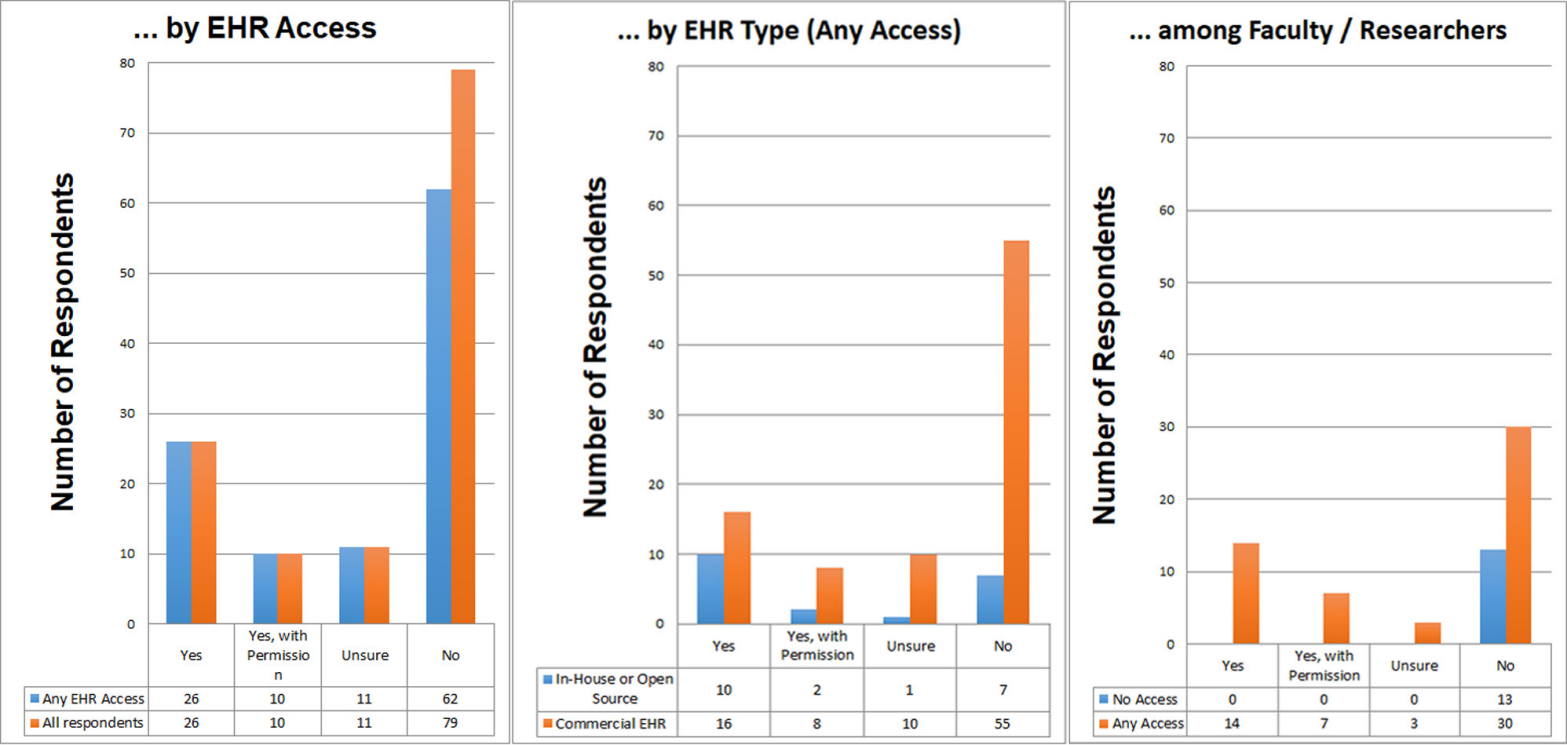
Characterization of Participants with EHR Access and the Ability to Publish EHR Findings in Usability Research.

**Figure 7:**
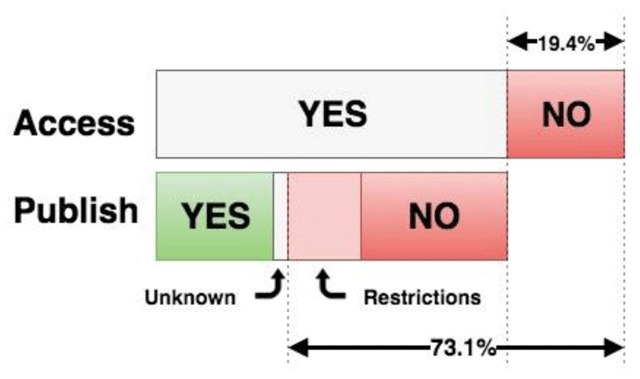
Breakdown of Faculty/Research Members with and without any EHR access (19.4 percent). Of those with access, ability to publish screenshots is shown.

Levels of publication censorship were further stratified by EHR type by comparing participants with commercial EHR systems to those with in-house or open source systems (Figure 6). Subgroup analysis of faculty members and researchers revealed that 72 percent had EHR access for usability and/or research purposes, but of those, fewer than one-in-three (29 percent) could freely publish screenshots without any vendor approval process. In this cohort, 15 percent reported possible publication with permission, 6 percent were unsure about publication restrictions, and exactly half of respondents reported that publication of screenshots was not allowed whatsoever (Figure 7).

## Discussion

Our survey targeted clinical informatics professionals, including faculty members and researchers involved with EHR usability-focused work. We sought to assess both levels of EHR access and the ability to publish results, neither of which have been previously quantified in the informatics literature among non-clinical users. Our survey sample represents prominent informatics roles (practicing clinicians, research scientists, and industry professionals), varied geographic locations (all major U.S. regions, with a majority from the Northeast, as well as international participants), and various EHR types (commercial vendor-based systems, in-house systems, and open source systems). Among this diverse sample, roughly one in ten reported no EHR access; however, rates were notably higher among faculty and researchers, with one in five reporting no access whatsoever. Among faculty members and researchers, 72 percent could access the EHR specifically for usability and/or research purposes, but of those, only one in three could freely publish screenshots while half could not publish such data at all. Across users from all roles, only one in five reported the ability to publish screenshots freely without restrictions.

Our work addresses an important gap in understanding levels of EHR access. Previous research has demonstrated wide variation in levels of EHR access among medical students, ranging from no-access to read-only access to full-access [6]. Tiwari and Kumar explored the use of algorithm-based permissions for users seeking access to patient-level EHR access by role, but their work did not seek to quantify current levels of EHR access among users across various roles [22]. Alassia et al. examined the implementation of a restricted-access EHR system which required justification criteria via dropdown menu or free-text entry for users to access patient-level data if they did not meet pre-specified login criteria [23]. While one possible justification criterion was “Health Informatics,” they found that for 20M+ logins, 85 percent did not require justification. Their work provided important data for *why* their users sought to access the EHR, they did not report or quantify current levels of EHR access by user role, especially among informatics professionals.

It is no surprise that over 70 percent of respondents reported some level of access to a commercial EHR, which aligns with the growing landscape of digitized health care systems dominated by commercial EHR vendors. While 15 percent of respondents use an in-house or open-source EHR, a non-trivial percentage, this represents institutions where access and publication rights are often most permissive; overall this percentage is proportionally shrinking and will likely reach near zero percent. We suspect it is only a matter of time before nearly all institutions still using homegrown systems transition to a vendor based EHR. Of participants in our survey who currently have access to a commercial EHR, fewer than 15 percent of those respondents reported the ability to publish their research without censorship of any kind. In contrast, roughly 50 percent of those with open source or in-house EHR systems had those same restrictions. While less censorship exists among users of open-source or in-house products, and these EHR systems are less common, the presence of any censorship nonetheless reflects institutional reluctance to share the same information as vendors.

Our data demonstrates moderate levels of limited access among faculty members/researchers (three in four can access the EHR for usability or research purposes). In other words, one in four faculty members/researchers lack EHR access at their institution for usability or research purposes, which alone suggests an unacceptable degree of academic restriction. Furthermore, notable publication limitations exist among this cohort, with less than 50 percent able to publish freely, including screenshots. The result of this level of censorship is that a vast majority of scientists researching EHR usability are either prevented from publishing screenshots altogether or must first obtain vendor permission, thus impeding the free dialogue necessary in communities of investigation.

We also observe apparent institutional variation in levels of access. Among participants whose institution used a commercial EHR product, nearly two-thirds of participants reported some level of EHR access. One-third had no EHR access whatsoever, raising concern that the problem of no-access among researchers may worsen as vendor-based systems become more ubiquitous and replace in-house systems. This raises the question of whether access privileges reflect EHR vendor policy or institutional policy. We suspect the latter, though it is worth exploring the extent to which contractual arrangements differ between vendors and different institutions. For instance, if some institutions are charged for the number of users with EHR access, this could incentivize institutions to limit EHR access among non-clinical faculty members and researchers.

We expected that the surveyed professionals would have some degree of limitation in terms of accessing EHRs for research purposes and of freely publishing their work; nonetheless, the discouraging results far exceeded our expectations. We did not expect access rates to be only 80 percent and certainly expected that more than 27 percent of respondents could freely publish screenshots (Figure 7). This paper supports the notion that low levels of EHR access among researchers negatively affects the optimization of EHR usability and, in turn, patient safety.

Based on our findings, we argue that by increasing levels of EHR access among non-clinical users—provided this is done carefully such that personal health information is not disclosed to users who do not need access to it—organizations and vendors could more rapidly optimize their user interfaces and user satisfaction. By allowing more eyes to view the same screens and more hands to “tinker in the same playground,” usability would advance at a faster rate. The following example is illustrative: a prominent non-clinical, health IT researcher personally appealed to the CEO of one commercial vendor “for permission to publish screenshots in a student’s master’s thesis…and was told no” [20]. In such a protective culture, how will the areas of implementation science, usability, and patient safety further advance? Our data suggest a need for changes in culture to embrace a more spirited exchange.

There have been numerous examples of medical errors attributed to EHR usability, including a well-known example of a patient who suffered a seizure after receiving a 39-fold overdose of an antibiotic [24]. These issues make headlines when tragedy strikes, but we fear they will continue until definitive changes are made. Challenges related to EHR usability can be addressed by investing the time and effort necessary for research and rigorous testing. Optimization in the areas of visualization, user experience, and user-centered design is key to empowering clinical end-users who must integrate EHR data into real-time decisions. Thus, in addition to technical expertise, EHR design requires an understanding of the end-users, their goals, and their workflow [2]. We argue that (1) lack of EHR access makes many critical EHR usability research activities impossible to conduct, and (2) publication censorship, especially regarding screenshots, means that even those usability studies which *can* be conducted may not have the impact they otherwise would. As a consequence, innovation can be stifled.

It is worth noting, however, that the currents may already be shifting. Commercial EHR vendors have expressed more willingness to cooperate with broader initiatives, including Apple’s prominent push to allow patients to view their own health data on their smartphone [25]. As the EHR landscape shifts, there is growing excitement for patient and provider-facing applications via the open standards-based platform known as SMART on FHIR. Given that usability and user-centered design are crucial to success in the application development space, vendors may find themselves under increasing scrutiny if the usability of applications which integrate with the EHR significantly outpaces that of the EHR itself, which will remain central to daily clinical work.

We join Sittig et al. [26] and others who have made previous calls for action and cooperation among the various stakeholders in health IT—vendors, clinicians, policy makers, and the informatics community—and we provide compelling new data to describe the extent of this problem. Specific recommendations related to usability include:

1. Loosening or abolishing policies that prevent researchers from publishing findings of EHR usability studies
2. Expanding EHR access to non-clinical researchers and scientists, possibly by increasing access to a robust EHR training environment that mirrors the actual clinical EHR at a given institution (thereby preserving patient confidentiality)
3. Mandating that screenshots and images from EHR systems be freely publishable without restrictions from copyright or trade secret constraints
4. Enforcing existing user-centered design principles more stringently (Table 2).

**Table 2:** Recommendations for Change.

Safety Area	EHR Users	Health Care Organization	EHR Vendors	Government Regulators

**EHR Accessibility**	Vocalize needs (both clinical and non-clinical users) Provide feedback to improve user training Participate in contract negotiations Participate in usability testing	Advocate for full access to an EHR training environment for researchers and scientists Include clinical and non-clinical informatics experts on institutional committees that regularly examine EHR use and challenges (i.e. multi-disciplinary team)	Provide a robust EHR training environment for non-clinical use that mirrors the actual EHR and can be freely accessed for usability research Partner with academic institutions to provide funding for usability research	Standardize contractual framework with vendors that demolishes “gag clauses” Support the rights for training and accessibility for non-clinical researchers
**EHR Knowledge Dissemination**	Participate in contract negotiations Advocate for uncensored dissemination of EHR study findings, including screenshots	Develop internal reporting system for usability issues, including medical errors in the EHR Provide support to openly publish EHR bugs or pain points Negotiate an agreement with vendors that allows for the dissemination of screenshots and key safety-related aspects of the EHR	Collaborate with other vendors to create industry groups that meet regularly to discuss common EHR problems and solutions Enable external publishing of EHR screenshots to improve overall usability Define “confidential” information, such as source code, design, databases etc.	Establish transparency requirements related to costs and performance of EHR systems Standardize contractual framework with vendors Protect academic/research rights to explore EHR design and functionality and to publicly disseminate findings Establish an EHR Safety Reporting System to which all ONC-certified vendors must adhere and enforce contractually

EHR vendors should be encouraged to support these changes, viewing them as part of a successful business model. Improving product usability will result in more satisfied users and improved patient outcomes; furthermore, customers will be more loyal after seeing better returns on their investments [2]. Open and free publication policies will also encourage innovation and speed adoption of best practices. The health care industry is a uniquely difficult environment for usability design, and the life-or-death impact of such designs makes restrictions that limit free and open evaluations of the EHR especially problematic.

### EHRs Should Fly

It is worthwhile to present a comparison to the aviation industry, which has similar profiles of risk, complexity, regulatory oversight, and technological integration. Despite fierce competition, multiple hardware vendors, software vendors, and airframe manufacturers have formed industry groups to regularly sanction safety conventions. Furthermore, the Federal Aviation Administration (FAA) has been regulating aviation for over twenty-five years by operating the Aviation Safety Reporting System, which issues safety alerts and assesses airworthiness [2,27]. The FAA has directly shaped aviation technology safety and usability through policy guidelines. This cooperation and transparency have helped the industry achieve high levels of safety despite high levels of risk and complexity.

Compared to the aviation industry, health care lags behind. While EHRs first penetrated health care over a decade ago, only in the last three years did the ONC implement similar practices for EHRs. In fact, many EHR vendors do not even comply with ONC policies, yet they maintain adherence certification nonetheless [27]. What will it take for our digital health system to achieve the safety profile of the aviation industry? If usability researchers have limited access to EHR systems and are not able to disseminate the results of their research fully, and if vendor adherence to regulation cannot be ensured, then EHR systems will continue to put patients at risk.

### Limitations

One of the study limitations is the low response rate to the survey invitation, as a higher response would have provided more statistical power. Nonetheless, the sample does include representation from all major U.S. geographic regions and internationally. Another study limitation was the number of questions included in the survey. Aiming for a high response rate, the authors condensed the survey questions to avoid burdening participants with a time-consuming, question-intense survey.

Lastly, the survey was distributed to informatics professionals through a professional society contact list which could limit the generalizability of the study’s results. Nonetheless, the authors also acknowledge this limitation as a study strength: if highly engaged and educated professionals with abundant resources do not have full access to EHRs nor the ability to freely publish their research, it is very unlikely that their counterparts outside of such prestigious societies have higher levels of access. These barriers could be overcome by conducting a similar survey that is disseminated through academic channels rather than a professional society, which might decrease reporting bias and increase generalizability.

### Future Directions

More investigation is needed to better understand the relationship between EHR usability and industrial, institutional, contractual, legal, and logistical factors. To improve EHR research, more transparency is needed regarding contract terms that health care organizations adopt. Currently, there are no policy standards regarding EHR accessibility, which prevents some researchers from viewing the EHR, and variation exists surrounding publication of usability research. Further work is needed to explore whether strategies such as increasing contractual transparency, expanding EHR access among non-clinicians, and liberalizing screenshot restrictions leads to improvements in EHR usability and patient safety.

## Conclusion

In summary, this study offers insight into current patterns of EHR accessibility among the informatics community. Our results demonstrate opportunities for improvement by increasing levels of EHR access for non-clinical researchers, creating a culture of discourse and innovation, and removing publication restrictions related to usability and evaluation. Health IT has the unique opportunity to drive advances in safety, quality, and satisfaction, but these advances require the cooperation of various stakeholders. It is time we all come together towards this shared goal.
